# Effects of homologous and heterologous rich platelets plasma, compared to poor platelets plasma, on cutaneous healing of rabbits^1^

**DOI:** 10.1590/s0102-865020200100000006

**Published:** 2020-11-23

**Authors:** Raquel de Oliveira Meira, Daniel Nogueira Mendes Braga, Leni Safira Gonçalves Pinheiro, Izabela Ferreira Gontijo Amorim, Leonardo de Souza Vasconcellos, Luiz Ronaldo Alberti

**Affiliations:** I Fellow PhD degree, Graduate Program in Pathology, Universidade Federal de Minas Gerais (UFMG), and Instituto de Ensino e Pesquisa (IEP), Santa Casa de Belo Horizonte, Brazil. Conception and design of the study; acquisition, analysis and interpretation of data; manuscript preparation and writing.; II Graduate student, Department of Surgery, Medical School, UFMG, Belo Horizonte-MG, Brazil. Technical procedures, acquisition of data, manuscript preparation and writing.; III Fellow PhD degree, Teaching and Research Institute, IEP, Belo Horizonte-MG, Brazil. Technical procedures, acquisition of data.; IV Full Professor, Department of Pathology, Faculdade de Minas (FAMINAS), Belo Horizonte-MG, Brazil. Histopathological examinations.; V Associate Professor, Department of Clinical Pathology, Medical School, UFMG, Belo Horizonte-MG, Brazil. Substantive scientific and intellectual contributions to the study, technical procedures, critical revision, final approval.; VI Associate Professor, Department of Surgery, Medical School, UFMG and IEP, Belo Horizonte-MG, Brazil. Substantive scientific and intellectual contributions to the study, conception and design, final approval.

**Keywords:** Platelet-Rich Plasma, Wound Healing, Rabbits

## Abstract

**Purpose:**

To evaluate and compare the effects of homologous and heterologous PRP (Platelet-Rich Plasma) on the quality and speed of skin wound healing, compared to Poor Platelet Plasma (PPP).

**Methods:**

Twenty-one male adult rabbits were used; two for preparing homologous PRP, with the rest of them separated randomly in three groups, according to the treatment received: PPP - control (n=5), homologous PRP (n=7), heterologous (n=7). Excisional skin wounds were made on the back of the animals, for the application of homologous and heterologous PPP and PRP. At the 14th post-operative day (POD), the animals were subjected to a new wound, and the treatments were inverted. The wounds were evaluated macroscopically and histologically.

**Results:**

A larger percentage of scar retraction was observed on the group treated with heterologous PRP, compared to homologous PRP, at the third POD, an increase of 25.03% (p=0.01). No other statistically significant differences among treatments were observed. Among every group, skin healing was efficient, without local adverse effects.

**Conclusions:**

Heterologous PRP contributed with more tissue retraction at the beginning of the wound healing process. After this, there were no differences on the wound healing skin process treated with PRP or PPP. However, our findings suggest the presence of others plasmatic factors, besides platelets, which could also contribute to the wound healing process, and thus, should be further investigated.

## Introduction

Platelet rich plasma (PRP) has been widely studied as a new biological matrix, capable of accelerating the wound healing process on skin, muscle, bone, cartilage and even tendon lesions, with favorable results^1^ ^-^ ^9^. PRP is plasma with elevated platelet concentration, which may be obtained through processes of seriated centrifugation. Platelets possess growth factors (PDGF, TGF-alfa, TGF-β, VEGF, IGF-I, PDECGF and EGF), which are able to repair and generate tissues, acting through different processes, such as mitogenesis, angiogenesis, chemotaxis, proliferation, and cell and fibroblastic differentiation^1^ ^,^ ^2^.

Most studies have evaluated wound healing processes through experimental models, after treatment with autologous PRP, that is, a biological matrix made from plasma obtained from the animal itself. However, in situations where treatment requires a larger PRP amount, or the patient has his or her physiologic conditions compromised, it may not be possible to collect his own blood in enough amounts for autologous PRP obtention. In those situations, alternatives sources are suggested for PRP, as homologous PRP, that is obtained from other individuals, or even heterologous PRP, obtained from other species^2^ ^-^ ^5^.

Positive results have already been obtained with homologous PRP use on the treatment of inferior member lesions, among diabetic patients, as well as on use of the combination of homologous PRP and heterologous plasma, for the treatment of corneal ulcers, in rabbits^2^ ^-^ ^5^.

Considering that the majority of works on the literature studies autologous PRP and considering the importance of developing other biological matrices, which may be used for stimulating the biological process of tissue wound healing, this work has the objective of evaluating macroscopic and histologic parameters of skin wound healing, in rabbits, after treatment with homologous and heterologous PRP. In order to obtain a control group, plasma with low platelet concentration, named Platelet Poor Plasma (PPP), was applied to the wounds.

## Methods

The study has been conducted at the *Bioterium* of the Medical School from the Universidade Federal de Minas Gerais (UFMG), after approval from the Animal Research Ethics Committee (CEUA/UFMG), protocol 203/2017.

Twenty-one New Zealand male rabbits, with ages between four and five months and body weight of 2.5KG were used. The animals remained inside individual cages, receiving food and water *ad libitum* during the whole experiment, at temperatures of (23±1ºC), with controlled sunlight exposure (12h light/dark), and daily cage cleaning.

The study was divided in two steps of 14 days each. Two animals were used for homologous PRP extraction and the remaining were submitted to a surgical skin wound at their backs, on the right side, and then distributed into three groups, according to the treatment received for their wounds:

- Group 1 (G1), control, treated with PPP (n=5);
- Group 2 (G2), treated with homologous in the first step PRP (n=7) / G2/2 treated with heterologous PRP in the second step.
- Group 3 (G3), treated with heterologous in the first step PRP (n=7) / G3/2 treated with homologous PRP in the second step.

During the 14 post-operative days (POD) the animals were evaluated, with daily photographic register of the skin wound healing process.

After 19 days, the animals from groups G2 and G3 were submitted to a new surgical procedure, like the first step, but inverting the sides of the surgical wound region (left back), as well the type of plasma applied in each group. The animals that received homologous PRP in the first step were treated with heterologous PRP in the second step, and the group that received heterologous PRP in the first was treated with homologous PRP in the second step.

**Figure 1:**
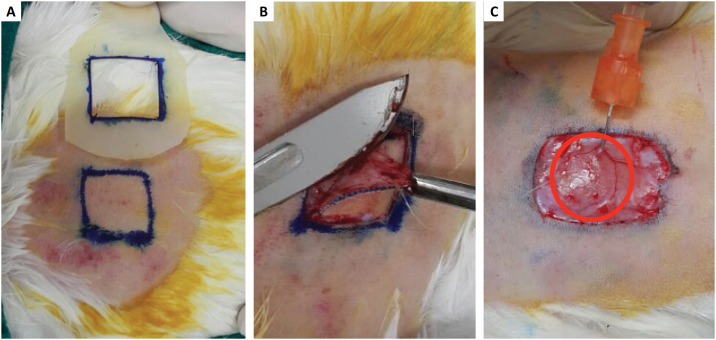
**a)** Mold of 4 cm² for standardization of the incisions, marking with pen on the dorsal surface of the animals, skull-caudal region of animal one of G1. **b)** Resection of skin fragment with the aid of anatomical forceps and scalpel blade, preserving the subcutaneous tissue, animal one from G1. **c)** Application of treatments to all animals in the central region, below the subcutaneous, the applied volume standardized in 0.4 ml (animal one from G1).

Every animal was properly sedated with ketamine (Ketalar®, Parke Davis Warner Lambert) at the dosage of 40mg/kg, combined with Xylazine (Xilazin®, Syntec), at the dosage of 7mg/kg, intramuscular. Hair removal was made with a naked blade and local asepsis (alcohol 70% solution and iodine), right after local anesthesia. A plastic mold measuring for square centimeters was prepared and sterilized, for wound size standardization, and then the surgical wound marking was made using a proper pen (Fig. 1).

After marking, the animals were submitted to the skin fragment excision, using a number 15 scalpel blade and surgical tissue forceps, preserving the subcutaneous tissue. Homologous PPP and PRP previously prepared were then applied using a 3ml syringe, at a volume of 0.4ml, at the central region of the wound, in the subcutaneous tissue. This process was repeated to each animal. At the end, every animal received one intramuscular dose of the analgesic Tramadol (0.5mg/kg per animal, single dose). After the plasma and analgesic application, the animals were submitted individually to photographs for registering to which group they belonged, date, as well as the first measures with rule and caliper of the surgical wound size.

Two other animals were used for preparing homologous PRP. The blood was obtained through abdominal vena cava puncture, with a vacuum system. Eight 3ml tubes with EDTA anticoagulant were filled, and then processed for PRP obtention. After basal platelet counting, these were submitted to the first centrifugation, at 800 rpm, in ambient temperatures, for eight minutes. After this, the supernatants were removed with a pipette, transferred to other tubes, and submitted to a new centrifugation, at 3000 rpm, for 15 minutes. The new supernatant, now poor in platelets, was removed and separated. This material was used on the Control Group - PPP. The remaining volume from each tube was agitated with vortex, for platelet suspension, resulting in the Homologous PRP. Platelet concentration increased from 405 × 10³/mm^3^ to 2.950 × 10³/mm^3^ after two centrifugations.

**Figure 2:**
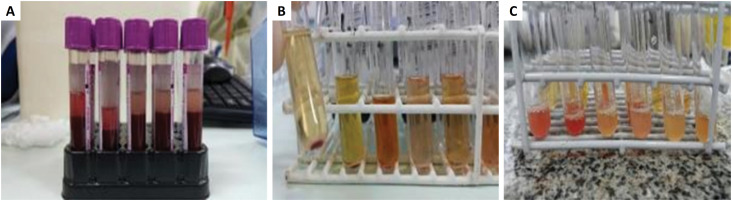
**A.** Tubes containing whole blood from the donor animal to produce the homologous PRP. **B.** Plasma after the 1st centrifugation. **C.** Plasma after the 2nd centrifugation, containing only PRP.

Heterologous plasma was obtained from a pool of eight EDTA tubes, from patients with high platelet concentration from the Clinical Pathology laboratory from the UFMG University Hospital. These samples were treated similarly to homologous PRP. Platelet concentration increased from 742 × 10³/mm^3^ (average between the eight tubes) before centrifugation to 3754 × 10³/mm^3^ after (Fig. 2).

**Figure 3:**
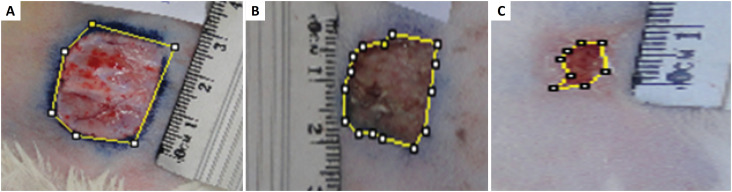
Measurement of the wound area of the first animal from group G2, with the software Image J. Outline of the area of scar retraction in G1 (PPP) **A)** 3rd POD, **B)** 7th POD, **C)** 14th POD.

Macroscopic analyses were conducted daily among every group, in two steps of 14 days each. The weight of the animals, measured with a scale and wound area dimensions, digitally measured, were registered. The wounds were observed daily and photographed using a 16.1 megapixels digital machine. Wound area calculation was done with the Image J software, available for free download (Fig. 3).

After defining wound areas at each observation moment (3^rd^, 7^th^ and 14^th^ POD), the percentage of scar retraction (Pc) was calculated, through the formula Pc=(Ai-Af) x100/Ai, with (Ai) standing for initial area, and (Af), final area, as suggested by Abegão *et al*.^5^.

Macroscopic parameters of the wound healing process as edema, exudates, hyperemia, granulation tissue and scabs, were measured according to the events observed at the 3^rd^, 7^th^ and 14^th^ post-operative days, and classified as absent (0) or present (1), for statistical analysis.

After the 37^th^ experiment day, a skin fragment of each wound was removed for histologic analysis. Sedation, anesthesia, hair removal, asepsis, and incision demarcation were done the same way as previously. Each fragment was placed over a piece of paper and introduced inside bottles containing 10% formalin.

These fragments were then sent to the Pathology Laboratory from the UFMG Medical School, where they were processed into paraffin blocks. These were cut into 5µm slices and then colored with hematoxylin-eosin (HE). The tissues were evaluated through optic microscopy, for the following parameters: ulcers, acanthosis, granulation tissue, scar tissue, and inflammatory infiltrate^6^. Every observation was made by the same observer.

Descriptive analysis and Kolmogorov Smirnov normality test were conducted for every result obtained by macroscopic analysis. The weight averages of the animals at the beginning and end of the experiments were determined and compared through paired t test. Percentages of scar retraction in each wound were compared among groups through non paired t tests. Categorical macroscopic variables observed in the wounds were also compiled, by calculating their occurrence percentages at the 3^rd^, 7^th^ and 14^th^ post-operative days.

The histopathologic analyses were classified on a semi-quantitative way, varying from: discrete, when less than 25 % of the fragment was affected; moderate, from 25% to 50%; and accentuated, when more than 50%. The probability tests used were Kruskal-Wallis e Mann- Whitney. All analysis was conducted with the program Bioestst. 5.0, with a significance level of 5% (p<0.05), and 95% confidence interval.

## Results

An increase on the weight average was observed among G1, G2 (p<0.01) and G3 (p<0.001), comparing initial and final weight. These statistically significant results show that there were no behavioral and nutritional changes which could compromise the wound healing process.

Ate the 3^rd^ POD, wound contraction percentage for the homologous group (G2) was significantly smaller than for the heterologous (G3) and homologous groups (p=0.03). However, there were no significant differences between G1 and G2 (p=0.20) and between G1 and G3 (p=0.32).

At the 7^th^ and 14^th^ POD the presented results did not show significant differences on wound contraction percentage among groups. At the 14^th^ POD, there was complete resolution of the healing process among every group (Table 1).

**Table 1:** Means and standard deviations of wound contraction percentages per group.

POSTOPERATIVE DAYS (POD)	Wound contraction (%)		
	G1 (n=5)	G2 -G2/2 (n=14)	G3- G3/2 (n=14)
3	33.946 ± 21.37	25.951 ± 17.243	38.358 ± 17.884
7	51.387 ± 16.177	47.256 ± 14.693	51.089 ± 17.356
14	99.994 ±0.006	99.997 ± 0.001	99.998 ± 0.002

G1: Control group; G2 –Homologous treatment group (1^st^ step); G2/2 - Homologous treatment group (2^nd^ step); G3 Heterologous treatment group (1^st^ step) – G3/2: Heterologous treatment group (2^nd^ step).

In the analyses comparing group G2 from the first step (homologous treatment) with G3/2 (heterologous treatment) from the second step, on the same animals, there were no statistically significant differences, as presented on Table 2.

**Table 2:** Means and standard deviations of wound contraction percentages per group (Homologous x Heterologous on the same animals).

POSTOPERATIVE DAYS (POD)	Wound contraction (%)		
	G2 (n=7)	G3/2 (n=7)	p value
3	31.10 ± 15,0	31.00 ± 20.12	0.49
7	48.03 ± 12.85	46.60 ± 20.94	0.43
14	99.99 ±0.00	99.99 ± 0.00	0.20

G2: First step homologous group; G3/2: Second step heterologous group. This table compared the evolution of wound contraction percentages with the use of different types of PRP treatment, on the same animals.

When G3 from the first step (heterologous treatment) was compared with G2/2 from the second step (homologous treatment), on the same animal, there was only significant difference at the 3^rd^ POD, with a favorable result for treatment with heterologous PRP (p<0.05), that is, G3 presented with faster contraction. However, on the following observation days (7^th^ and 14^th^), results also did not show significant differences, as shown by Table 3.

**Table 3:** Means and standard deviations of wound contraction percentages per group (Homologous x Heterologous on the same animals).

	Wound contraction (%)		
POSTOPERATIVE DAYS (POD)	G3 (n=7)	G2/2 (n=7)	p value
3	45.73 ± 12.27	20.70 ± 18.82	0.01
7	55.52 ± 12.91	46.47 ± 17.35	0.12
14	99.99 ±0.00	99.85 ± 0.37	0.17

G3: First step heterologous group; G2/2: Second step homologous group. This table compared the evolution of wound contraction percentages with the use of different types of PRP treatment on the same animals.

The observed categorical variables were edema, exudate, hyperemia, granulation tissue and scabs. The first three ones were more evident at the first three post-operative days. As time passed, granulation tissue and scabs became more prominent, as expected for the normal skin healing process.

Edema was largest at the third POD, progressively decreasing until the 7^th^ POD, having disappeared completely at the 14^th^ POD. The same happened for hyperemia. Exudate was observed the most at the 3^rd^ POD for control group, at the 3^rd^ and 7^th^ for homologous group (the most discrete), and at the 7^th^ POD for heterologous group.

The presence of granulation tissue was the largest at the 3^rd^ and 14^th^ POD for control and homologous groups and at the 14^th^ POD for heterologous group.

The presence of scabs was observed at every moment, for every group, however with the least intensity at the 14^th^ POD.

At the end of the experiment, the following aspects were observed by histopathological analysis.

### a) Group 1 (control):

**Figure 4:**
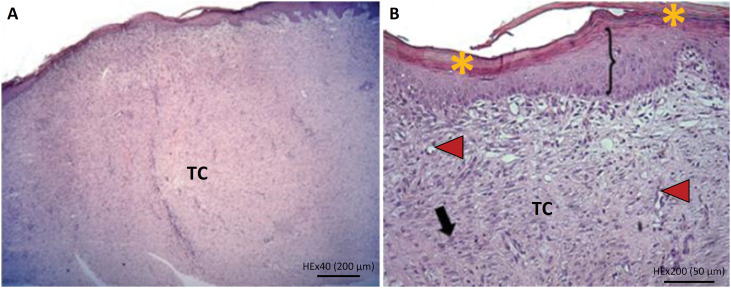
Histological rabbit skin cut, treated with poor platelet plasma (Control-G1). **A.** Panoramic view of cicatricial tissue (x40). **B.** Observe, in detail, the presence of acanthosis (*brackets*), and hyperkeratosis (*yellow asterisk*). Cicatricial tissue with fibroblasts (*black arrow*), and blood vessels (*red arrowhead*|) (x200).

- Epidermis: regenerative tissue and remodeling, with four animals presenting discrete acanthosis (<25%) and another with moderate acanthosis (≤ 50%);
- Superficial and deep dermis: presence of collagen, fibroblasts on three planes and some neovascularization. Tissue varying between granulation, conjunctive and cicatricial, with discrete and mixed inflammatory infiltrate (3/5) (Fig. 4).

### b) Group 2 (Homologous):

**Figure 5:**
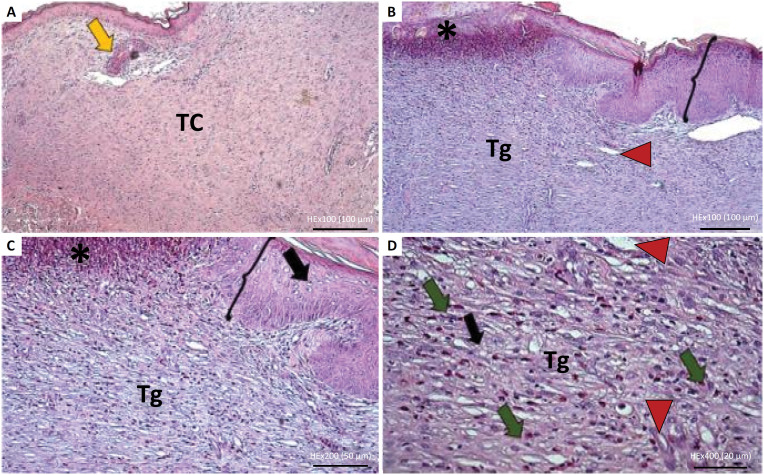
Histological rabbit skin cut, treated with homologous rich platelet plasma (G2). **(A)** Fibrous cicatricial tissue (TC) and skin attachment (*yellow skin*) (x100). **(B)** Ulcer with fibrinoid necrosis at the epidermis (*asterisk*) and area of acanthosis (*brackets*). Granulation tissue at the dermis (Tg), inflammatory infiltrate and neovascularization (*red arrowheads*) (x100). **(C)** The previous image with an ulceration area (*asterisk*) and acanthosis (*brackets*), with vacuolization of keratinocytes (*black arrows*). At the dermis there is granulation tissue (Tg) (x200). **(D)** Granulation tissue (Tg), with a moderate ammount of eosinophils diffusely, and neoformed blood vessels (*red arrow*) (x400).

- First step: epidermis was regenerative, with discrete acanthosis (<25%) to intense (>50%), with no ulcers observed. At the dermis, there were vascular neoformations, little cellularity and cicatricial tissue deposition, in 4/5 there was mixed type inflammatory infiltrate (<25%) (Fig. 5A).
- Second step: at the dermis of five animals there was ulcer with serosanguineous scabs. These presented at the superficial and deep dermis, neovascularization, fibroblast presence and fibrous conjunctive tissue (granulation tissue), besides mixed inflammatory infiltrate, varying from discrete (25%) to moderate (≤ 50%) with eosinophils predominance. In two rabbits, there was granulation tissues with ulcers. At the end, an animal presented scabs and conjunctive cicatricial tissue with discrete eosinophilia (Fig. 5 B-D).

### c) Group 3 (Heterologous):

**Figure 6:**
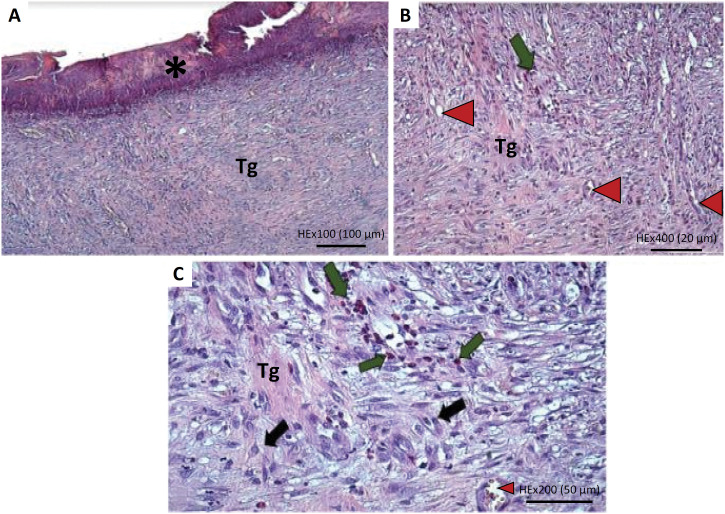
Histological rabbit skin cut, treated with heterologous rich platelet plasma (G3). **(A)** Ulcer with fibrinoid aspect through the epidermis (*asterisk*). Granulation tissue at the dermis (Tg) (x100). **(B)** Granulation tissue (Tg) and neovascularization (x100). **(C)** Granulation tissue (Tg) with discrete focal inflammatory infiltrate (*green arrow*), neovascularization (*red arrow*) and fibroblasts (*black arrow*) (x200).

- First step: six animals presented regenerative epidermis with acanthosis, varying from discrete (<25%) to intense (>50%), with none of the animals presenting ulcers. The dermis had a cicatricial aspect (Fig. 6A). A mixed and focal inflammatory infiltrate was identified in three of these animals; however, in one of them, there were extensive serous-cellular scabs, associated to the subjacent dermis and intense eosinophilic inflammatory infiltrate (>50%) (Fig. 6 B,C).
- Second step: four of the seven animals presented ulcer areas and serous-cellular scabs, around the epidermis. At the superficial and deep dermis, there was granulation tissue and among every animal from the group there was an inflammatory pattern, predominantly eosinophilic, varying from discrete (<25%) to moderate (≤ 50%).

**Figure 7:**
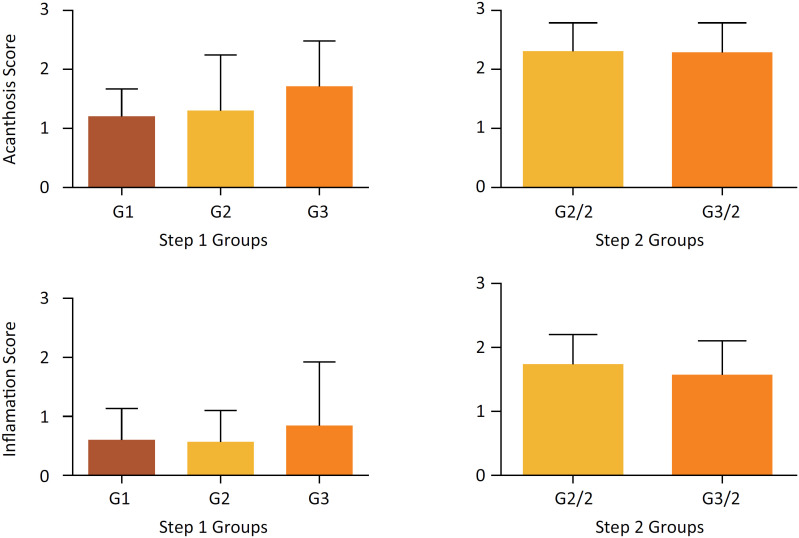
Degree of acanthosis and inflammatory infiltrate in the skin of rabbits submitted to Poor Platelet Plasma (G1, n=5), Homologous Platelet Rich Plasma (G2 and G2/2, n=14) and Heterologous Platelet Rich plasma (G3 and G 3/2, n=14), in the 1^st^ and 2^nd^ steps. Groups do not differ at the 5% probability level by the Kruskal-Wallis and Mann-Whitney test.

There were no statistically significant differences on the semi-quantitative evaluations for acanthosis and inflammatory infiltrate in both steps (Fig. 7).

## Discussion

One of the differentials of this study was using a control group with poor platelet plasma obtained from heterologous blood, rather than with 0.9% saline solution (NaCl 0.9%), as in most works in the literature. This was done as to eliminate the possibility of interference by other blood elements, besides platelets, in the healing process.

The kind of plasma applied to the animals of groups 2 and 3 was swapped. This process allowed duplication of the sample number and, most importantly, the reduction of possible interferences related to the biological variability bias among the animals. For data collection and information regarding the preparation and use of PRP, some guidelines were used as the articles from Barrionuevo *et al*. and Costa *et al*.^3^ ^,^ ^5^ ^,^ ^7^ ^,^ ^8^. The latter was the most used, using PRP in the treatment of medial ligament lesions in rabbits. Two centrifugations were done, the first one at 800 rpm for 8 minutes and the second, after supernatant removal, at 3200 rpm for 15 minutes^22^.

The results of platelet concentration were equivalent to other works in the literature, which report increased efficiency with platelet concentrations six times larger than normal. The PRP form used in this study was liquid, for being more practical, economically viable, easier to process and to equally distribute throughout the wound. The same PRP form was used by Vendramini *et al*.^8^ and confirmed by Kemper *et al*.^10^, who evaluated use on skin grafts.

Although no significant difference was observed for reduction of scar contraction, heterologous PRP behaved similarly to the autologous and homologous forms, as concluded by Barrionuevo *et al*.^3^ ^,^ ^5^. However, Ostavar¹¹, in his work with autologous PRP, reported significant difference between control group and treated, with significant lesion reduction.

In order to explain the lack of effect of PRP use in this study, there could be other plasmatic factors, not related to platelets, acting in the skin healing process. Another explanation could be tied to methodological differences. In this work, only one PRP application was done in the skin tissue. Bauer *et al*.¹², for instance, applied PRP on skin wounds in a single day, contrary to other studies which made successive applications. One successful example was the work by Santos *et al*.^9^, which used PRP applications every two or four days into chronic ulcers, resulting in a positive result for speed of wound healing retraction.

Barrionuevo *et al*.³ used three treatments in rabbit skin wounds: homologous PRP, autologous and heterologous and concluded there were no statistically significant differences.

However, Pazzini *et al*.^4^ found exudate presence for longer than the initial scarring phase, mainly in the control group, compared to the one treated with PRP, something that differs from this study, as this was observed for a shorter period of time in our study. Vendramini *et al*.² used grafts associated to autologous PRP gel, observing significant exudate presence in the treated group. The author linked this to a better cicatricial process in the inflammatory phase.

The study by Kemper *et al*.^10^ also used autologous and homologous grafts, with and without PRP, observing serous-sanguineous, non-purulent exudate presence, without any contamination. However, increased inflammation could be unfavorable to scarring, as it could increase edema, hyperemia, exudate and pain, generating post-operative complications and retarding the wound healing process.

Presence of granulation tissue was more evident as observation days passed, mainly among groups G2 – G2/2 and G3 – G3/2. There was no hyper granulation, reinforcing results from Abegão *et al*.^5^ and Ostavar *et al*.^11^ ^,^ ^15^.

The inflammatory infiltrate tends to reduce the most approaching the 14^th^ POD. According to Vendramini *et al*.², this happens from benefits of PRP use in neovascularization. The alternative sources used in this study do not seem have any adverse effect on this process.

Scabs were present through most of the observation days, and this was confirmed by histopathology. As noted by Costa *et al*.^16^, this is expected of the normal skin healing process.

Histological analysis demonstrated that the healing process occurred normally, with the presence of tissue reepithelization, presence of inflammatory cells, and neovascularization, the same as described by Barrionuevo *et al*.^3^ ^,^ ^5^.

Results from G1 presented remodeling and discrete to moderate acanthosis, besides discrete to mixed inflammatory infiltrate at 37^th^ POD. This result differed from Perches *et al*.^17^ ^,^ ^18^, who used PPP and PRP for treating corneal ulcers in rabbits, and observed a larger inflammatory infiltrate in the group treated with PRP, comparing to the control (30^th^ POD), suggesting a positive answer on the expression of matrix metalloproteins.

Findings from groups treated with PRP G2 and G3 at the first stage in were similar, presenting mild to intense regenerative acanthosis and absence of ulcers. In contrast, group PPP did not have the same result, suggesting a significant increase of the former with PRP use, as reported by Bauer *et al*.^12^, and Pazzini *et al*.^4^.

The scarring was similar, there were no adverse reactions, there were no observed changes in the healing time, and thus there was no evidence of compromise to the wound healing process brought by either homologous or heterologous PRP.

It is important to note that technique for obtaining and preparing the PRP must be aseptic to prevent any contamination that could compromise the quality and efficiency of the treatment^8^.

Clinical uses of PRP in tissues other than skin are also being widely studied, as described by Anitua *et al*.^19^ ^,^ ^20^, who used bone grafts to aid implantation process in dental and ophthalmic treatments, by Perches *et al*.^17^, who treated corneal ulcers in rabbits, as well as studies in more complex areas such as cardiovascular treatments described by Mörschbacher *et al*.^21^, who used PRP associated with stem cells to treat dilated cardiomyopathy in rabbits.

Further research is suggested regarding the use of PRP in skin healing, using the same standardization as this experiment, but with serial applications and a longer observation time in addition to other specific laboratory analyzes such as biochemical, morphometric and immunohistochemical analyzes. This could confirm the safety of using treatments obtained from plasmas of non-autologous origin, to increase their availability for routine hospital practices.

However, these findings suggest the presence of other plasmatic factors, besides platelets, which could also contribute to the wound healing process, and thus, deserve to be further investigated.

## Conclusion

Heterologous PRP contributed with more tissue retraction at the beginning of the wound healing process. In the other periods, there was no difference on the wound healing skin process treated with PRP or PPP.
